# Bibliography

**Published:** 1892-11

**Authors:** 


### Bibliography.

The Essentials of Histology, Descriptive and Practical, for the Use of Students. By E. A. Schäfer, F.R.S., Jodrell Professor of Physiology in University College, London, etc. Third edition, revised and enlarged. Lea Brothers & Co.: Philadelphia, 1892.

The fact that this book has reached a third edition is sufficient evidence that it has made a place for itself among the workers in histology. The author says in his preface that it "is written with the object of supplying the student with directions for the microscopical examination of tissues, . . . and intended to serve as an elementary text-book of histology."

The book is divided into forty-five lessons, beginning with the "Use of the Microscope, Examination of Common Objects." Each lesson is preceded by a practical description of the work necessary to bring the various tissues into view, with the reagents required and minute instruction as to their use. These are so clearly made that one with a limited knowledge of the microscope should be able to follow them without the aid of a teacher; but, this not being the intention, they become doubly valuable when superadded to class illustrations.

These directions are followed by a general description of the tissue or tissues under examination.

The portion devoted to the preparation of sections of teeth is not as detailed in statement as in most of the other lessons. It would be impossible to follow the modes prescribed and succeed in securing satisfactory results. Nothing is gained in histological accuracy by advising the student that "it is better to purchase"

the specimens used, as "the preparation is difficult and tedious." This, in our judgment, is not the way to study the tissues of the teeth. What is needed is careful preparations of *perfectly fresh specimens*, and, while the work is tedious, the results will be satisfactory in proportion to the care with which this is done and the shortness of time which is allowed to intervene between extraction and mounting.

The book is worthy of introduction in all colleges possessing a well-equipped histological laboratory.

Questions and Answers on Dental Pathology and Therapeutics, Dental Embryology, Hygiene, and Care of Children's Teeth. By Gustavus North, A.M., D.D.S., Professor of Dental Pathology, etc., U. S. Dental College, Chicago, Ill.

This little book of sixty-eight pages has been prepared, it is presumed, to be an aid to the study of the various subjects named in the title, but exactly why a teacher should regard it in this light it is difficult to determine. All books of this character, it seems to us, lead directly to superficial work, and, while they may aid in cramming certain leading points, inevitably weaken the ability to assimilate scientific facts in their completeness.

The answers to the questions in some instances are, in our opinion, misleading, as, for instance, in the portion devoted to dentition and dental caries.

